# Biases in butterfly survey effort and patterns of protocol used across biogeographic regions

**DOI:** 10.1002/eap.70326

**Published:** 2026-10-04

**Authors:** Joseph Izang Ibrahim, Sofie Spatharis, Rob Cooke, David B. Roy, Oskar Brattström

**Affiliations:** ^1^ School of Biodiversity, One Health and Veterinary Medicine, University of Glasgow Glasgow UK; ^2^ A. P. Leventis Ornithological Research Institute, University of Jos Centre of Excellence in Ornithological Training and Research Laminga Nigeria; ^3^ UK Centre for Ecology and Hydrology Wallingford UK; ^4^ Centre for Ecology & Conservation University of Exeter Falmouth UK; ^5^ African Butterfly Research Institute Nairobi Kenya

**Keywords:** biodiversity assessment, butterfly conservation, butterfly monitoring, butterfly sampling methods, global butterfly index, global research effort, insect conservation, monitoring trends in Lepidoptera

## Abstract

Butterflies are widely recognized as effective bioindicators due to their short life cycles, close associations with specific host plants, and rapid responses to environmental change. They are comparatively easier to identify than many other insect groups, making them valuable for biodiversity monitoring. Despite this, there is no consensus or standardized guidelines on butterfly monitoring protocols in many regions of the world. This lack of standardization can compromise data quality, limit comparability across studies, lead to inefficient use of resources, and ultimately weaken our capacity to track and understand butterfly population trends at regional and global scales. Here, we systematically reviewed 435 published studies on butterfly surveys to understand: (1) butterfly survey effort across biogeographic regions and identify areas in need of monitoring and method development, (2) examine historic trends in the use of butterfly survey protocols, and (3) assess whether studies explicitly justify their choice of protocol. Our results show that butterfly survey efforts have increased over time across all biogeographic regions but remain strongly biased towards temperate regions of the Global North, particularly the Palearctic and Nearctic. We found high diversity in protocol terminology (*n* = 29 protocol names), and wide use of different protocols (*n* = 7 protocol types). Line transects were the most frequently used protocol type, with at least 50% of studies using this protocol at any point in time since 1970. However, the use of alternative protocol types has surged in recent years. Despite this diversity, explicit justification for protocol choice was generally lacking. Together, these patterns show major gaps in monitoring coverage and inconsistency in methodological practice without justification. Overall, our findings demonstrate the need to intensify butterfly monitoring in underrepresented regions, particularly the Afrotropical, Neotropical, and Papuan regions, as well as highlighting the need for more systematic, comparative evaluations of survey protocols across different biomes to support evidence‐based protocol selection, facilitating robust cross‐study comparisons and informing global butterfly conservation efforts.

## INTRODUCTION

Insects are the most speciose animal group, with a key role in ecosystem function that is critical to human well‐being (Noriega et al., 2018). Among insects, butterflies stand out as the most comprehensively monitored group (Thomas, 2005), largely because of the availability of practical survey methods and their sensitivity to environmental change. Compared to other insects, butterflies have relatively well‐documented life histories and have close associations with specific or a limited suite of host plants (Sparrow et al., 1994). These characteristics contribute to the wide use of butterflies as indicators for assessing biodiversity change and informing conservation policy (Edwards et al., 2025; Roy et al., 2015; Thomas, 2005).

Recent studies show that butterfly populations, like many other taxa, are undergoing significant declines at global, regional, and local scales (e.g., Edwards et al., 2025; Plummer et al., 2023; van Deynze et al., 2024; Warren et al., 2021). For example, analysis of over 12 million butterfly records in the United States revealed an average decline of approximately 22% in butterfly abundance between 2000 and 2020, with one‐third of species showing significant decreases, many exceeding 50% loss (Edwards et al., 2025). These declines have drawn interest from the media, policymakers, and the public, prompting widespread conservation efforts.

Protocol standardization is a fundamental component of effective conservation efforts, because it provides evidence for direct conservation action (Montgomery et al., 2021). However, the multitude of available survey protocols can pose challenges for generating high‐quality, comparable data necessary for assessments across regions and timescales. While variation in protocols can complicate comparisons (Elphick, 2008), such differences are less problematic if they produce robust and unbiased abundance estimates (Jetz et al., 2019). Factors such as site selection, habitat heterogeneity, and observer experience could influence detectability and, ultimately, population estimates (Pescott et al., 2025; Schmidt et al., 2023). However, the survey protocol sits at the center of these influences, shaping how data are collected and interpreted, and therefore remains a fundamental determinant of monitoring reliability. This highlights the importance of selecting appropriate survey protocols that detect reliable population estimates over time rather than relying on convenience or tradition. Moreover, for butterflies to serve as effective biodiversity indicators, monitoring must employ protocols capable of detecting either specific target species or a broad range of species where biases in survey methods are well understood (Viliani et al., 2023) and go beyond simple presence–absence data to provide a sensitive measure of population change (Elphick, 2008). These considerations should be accounted for when comparing trends between different monitoring schemes (Gonzalez et al., 2023). Standardized approaches are essential for generating robust, comparable data across spatial and temporal scales, yet current practices vary widely in methodology, effort, and reporting (Kühl et al., 2020). This variability can introduce biases in butterfly assessments and hinders the capacity to detect long‐term trends or evaluate conservation outcomes at the regional and global levels. Furthermore, the global distribution of monitoring efforts is uneven, with some regions receiving disproportionate attention relative to their biodiversity (Hughes et al., 2021; Riva et al., 2026). Understanding these patterns and methodological choices is important for identifying gaps and biases in monitoring effort, improving comparability among monitoring schemes, and supporting evidence‐based conservation decision‐making (Lindenmayer & Likens, 2009). Such knowledge is also critical for recent efforts to develop a Global Butterfly Index (e.g., Riva et al., 2026), which integrates data from multiple monitoring schemes worldwide to assess population trends and support conservation planning at global scales. Against this backdrop, we systematically review the development and historic application of butterfly survey protocols across different biogeographic regions of the world to identify gaps, biases, and opportunities for harmonization. To achieve these goals, we address five specific questions:

1. What are the historic trends in published butterfly survey efforts across global biogeographic regions?
2. How have efforts been distributed in relation to regional butterfly species diversity and the size of biogeographical areas?
3. Has the choice of butterfly survey protocols changed through time?
4. Is the use of survey protocols clearly justified within studies?
5. Does the choice of protocol depend on the type of research questions asked, the biogeographical region, and/or the target focal group (e.g., general butterfly biodiversity, functional groups of species, single species populations)?

## METHODS

All data processing and analyses were carried out in R version 4.3.1 (R Core Team, 2023) and plots were prepared using *ggplot2* version 3.5.1 (Wickham, 2016).

### Bibliography search, studies screening, and selection criteria for inclusion

We performed a systematic literature search using Web of Science (Martín‐Martín et al., 2018) on 18 May 2024 using a search string combining 24 relevant keywords and applying Boolean logic within the titles, abstracts, and keywords, covering the complete time up to May 2024. Our search string (Appendix S1: Box S1) contained all keywords directly linked to our research topic; specifically, it included all the terms and synonyms to the study organisms and survey protocol terms relevant to the research area (Appendix S1: Box S1). The search generated a list of 1631 studies that went through our identification, screening, and inclusion procedures following the PRISMA guidelines for systematic review (Page et al., 2021) (Figure 1). We sorted and de‐duplicated the extracted list (duplicate records = 68) using the “distinct” function (by exact matching of study titles) in the *dplyr* package (v. 1.1.4) (Wickham et al., 2023). To streamline our review, we then filtered the remaining studies based on their titles and abstracts using the “filter” function in the *dplyr* package, followed by manual scanning of titles of all studies. Studies that did not have the word “butterfly” or its synonyms (see our search string) in the abstract and title were dropped (*n* = 878; Figure 1). We retrieved the full publications of the relevant studies from the libraries of the University of Glasgow and the UK Centre for Ecology and Hydrology. Any sources not available through these libraries were then searched for through other means (e.g., Google Scholar, ResearchGate, and targeted web searches). A small number of publications could not be accessed (*n* = 21; Figure 1) and were excluded from further analysis. Accessible studies were manually screened against a set of exclusion criteria presented below (numbers in brackets are studies that did not meet that criterion):

1. Studies not published in English (*n* = 151). This criterion was applied to increase the review speed and address the lack of expertise in other languages among the authors. Although this can introduce a bias (Konno et al., 2020), we followed recommendations to exclude other languages during the eligibility step where necessary, rather than the screening step (Pieper & Puljak, 2021).
2. Studies that do not report the use of a butterfly survey protocol (*n* = 87).
3. Studies that were not published in a scientific journal (*n* = 37). We restricted our review to peer‐reviewed literature to ensure a clearly defined, reproducible, and systematically searchable evidence base across regions and time periods. We acknowledge that this approach may underrepresent some butterfly monitoring efforts, particularly those conducted by local nongovernmental organizations (NGOs), conservation organizations, and government agencies that are documented primarily in gray literature.

From the 435 published studies that met our inclusion criteria (Ibrahim et al., 2026), we extracted the following information by reading the full text of each publication:

1. Research question: Studies were grouped into six categories based on what type of research question was addressed using monitoring data (general ecology, ecosystem services, population monitoring, effects of habitat disturbance, protocol development, and effects of climate change).
2. Country of study.
3. Estimated duration of study (in months).
4. Study focus group: Whether the study is targeted at butterfly biodiversity more widely, a selected group of species (e.g., frugivorous butterflies), or a single species.
5. Survey protocol type used for data collection (Table 1). We grouped studies according to the fundamental sampling logic that underpinned each protocol. Protocols that involved observers walking a predefined route while counting butterflies were collectively grouped as line transects. Protocols where the observer remained stationary and recorded butterflies for a fixed period were classified as point counts. When the observer moved through a site for a set amount of time to document species, we grouped these as timed surveys. Approaches that generated counts or checklists within a clearly defined area but did not specify a time component were considered area surveys. We treated records arising from incidental sightings, photographic submissions, or museum specimens as opportunistic observations. All protocols using Van Someren‐Rydon traps or similar baited trap designs were aggregated under bait trapping, while protocols based primarily on catching butterflies with sweep nets were grouped as hand netting.
6. Protocol justification: If the study explicitly justified the choice of survey protocol used, and the rationale for why it was suitable.

Studies that employed more than one field protocol were disaggregated, with each protocol recorded as a distinct entry under the same study. Studies spanning more than one country or addressing multiple research questions were treated similarly to ensure that each unique methodological or contextual component was represented as an independent entry in the dataset. We further grouped studies into biogeographic regions, following the six‐tier classification used by Larsen (2005). We chose this rather simplified classification because it provided recent estimates of the number of described butterfly species in each region, which enabled comparisons of monitoring efforts to regional butterfly species diversity. We additionally obtained the total land area of each biogeographic region (Loidi & Vynokurov, 2024).

**FIGURE 1:**
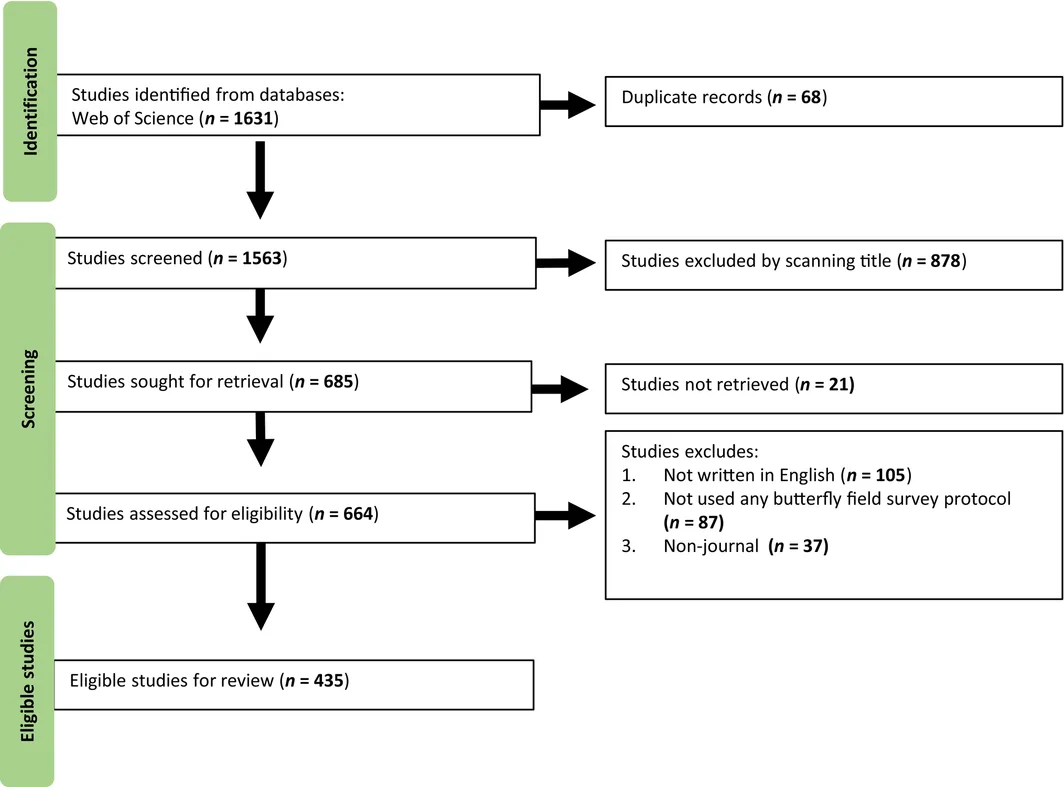
Visual representation of studies identified, screened, processed, and excluded based on a set of criteria for systematic review (Page et al., 2021).

**TABLE 1:** Classification of butterfly survey protocols reported across the studies into broader survey categories.

Broader classification	Reported protocol
Area survey (52)	Area survey
	Checklist surveillance
	Garden count
	Garden survey
	Microhabitat survey
	Plot counting
	Roost counting
Bait trapping (60)	Bait trapping
	Mark‐release‐recapture (using bait traps)
	Trapping
	Van Someren–Rydon trapping
Hand netting (64)	Collection
	Hand netting
	Insect net
	Mark‐release‐recapture (using hand net)
Line transects (298)	Driving count
	Distance sampling (based on transect)
	Line transects
	Pollard walks
	Road count
Opportunistic observation (13)	Opportunistic observation
Point count (23)	Circle count
	Focal observation
	Hawk watch census
	Point count
Timed survey (19)	Rule‐based stopping protocol
	Time‐constrained survey
	Timed survey
	Zig zag walk

*Note*: Brackets show the number of studies (*n*) out of a total of 529 protocol uses reported from the 435 published studies used in this review. Protocols are arranged in alphabetical order.

### Data analysis

To understand the historic trend in butterfly survey studies in different biogeographic regions, we calculated the annual cumulative number of months of butterfly survey studies for each region using the “cumsum” function. To assess the relative representation of the six biogeographic regions, we first computed the proportion of number of months of butterfly surveys in a region relative to the total number of months of butterfly survey for all regions. We then repeated these calculations for the number of described butterfly species per region and the total area of each region. Finally, we computed the difference between the month of survey proportion and species proportion, and the geographic area of each region (e.g., the Palearctic had 42% of studies but 11% of total butterfly species and 41% of the global terrestrial surface area). Positive values indicated that a region was overrepresented in butterfly survey research relative to its species diversity or land area, while negative values suggested underrepresentation (Troudet et al., 2017).

To investigate how the use of protocols has changed over time, we calculated the proportion of all studies that used each protocol per year. To explore the degree of justification, we then calculated the proportion of studies that justified the choice of protocol. We calculated both an average justification level for all types of protocol and individual levels for each type of protocol. For this analysis, we removed all studies that used data from any national monitoring schemes (*n* = 74) as the justification (or lack of it) was assumed to be undertaken at the point of setting up such schemes. Likewise, papers using secondary data were not included as the justification was assumed to have been made in the original study (*n* = 7).

To investigate the factors influencing the choice of butterfly survey protocol, we fitted a Bayesian multinomial logistic regression model using the “brm” function in the *brms* package version 2.21.0 (Bürkner, 2017). We used a categorical family to model the multinomial nature of the response variable (aggregated butterfly survey protocol categories, *n* = 7), with research question, estimated study duration (months), study focal group, and biogeographic region included as explanatory variables. We used the stepwise function (Genell et al., 2010) to select the best model based on Akaike information criterion (AIC) (Bozdogan, 1987). Coefficients derived from the analysis were used to compute the likelihood of selecting each butterfly survey protocol.

## RESULTS

### Overview of key findings

The 435 published studies compiled for this review (and that passed all eligibility criteria; Figure 1) were based on either original field data, reused data from earlier research, or data from long‐term butterfly monitoring schemes. The first retrieved study reporting on the use of a field protocol was published in 1979, 3 years after the first systematic butterfly monitoring project was set up in the United Kingdom (Pollard, 1977). Spatially, we recorded studies from 69 countries, representing all six biogeographical regions (Figure 2). However, six countries (United States of America, United Kingdom, Brazil, India, Indonesia, and Spain—ordered by contribution) accounted for over 50% of the global studies. More broadly, the Palaearctic region had the highest proportion of published studies (42%), followed by the Nearctic (21%), Oriental (15%), Neotropical (14%), Afrotropical (6%), and Papuan (2%) regions. We recorded a high diversity of names for butterfly survey protocols (*n* = 29), partly attributed to the numerous synonyms and variations in nomenclature depending on authors and region. We aggregated similar protocols into seven broader categories (Table 1).

**FIGURE 2:**
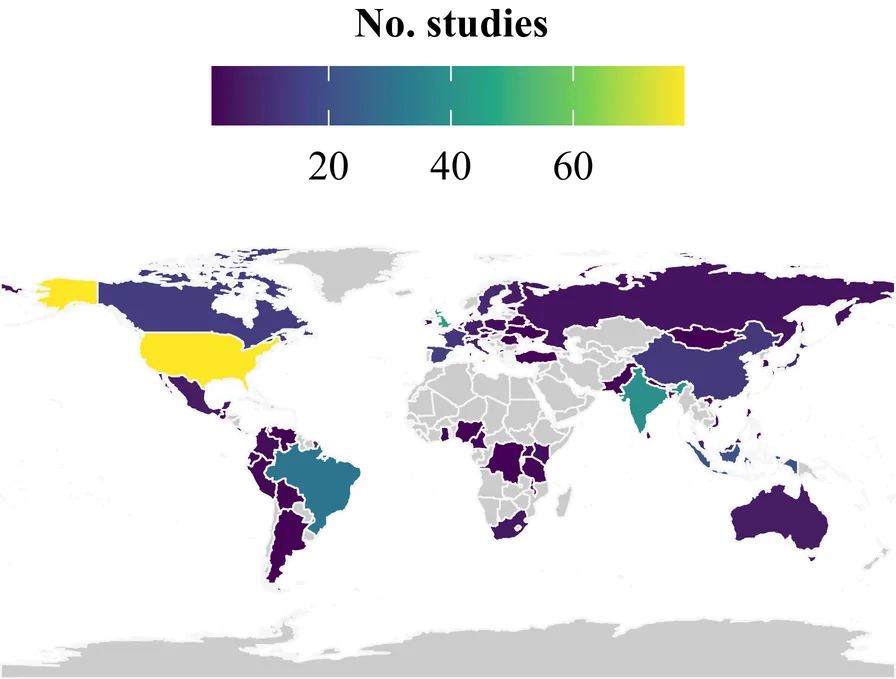
Geographic distribution of studies compiled by our systematic literature review. The color gradient represents the number of studies per country, while countries in gray were not represented in any of the studies.

### Global pattern and trends in butterfly survey effort

The temporal progression in the cumulative number of months of butterfly surveys using butterfly survey protocols (hereafter, butterfly survey effort) across the six biogeographic regions (Figure 3A; Appendix S1: Figure S1) showed some marked differences (Figure 3B; Appendix S1: Figure S1). In regions with a longer history of butterfly monitoring (Palearctic and Nearctic), the progression was initially slow following the first butterfly survey in those regions (first published butterfly survey in 1979 in the Palaearctic region, and 1981 in the Nearctic). However, there was a later surge in butterfly survey effort in these regions during the late 1990s, followed by a further increased rate in the Palearctic around 2010 (see Figure 3B; Appendix S1: Figure S1). In regions with a more recent onset of butterfly monitoring, the Neotropical (first published survey in 1991) and Oriental regions (1997), there has been a strong increase in published survey effort over the last decade, and the total numbers of butterfly surveys from these two regions are now close to those from the Nearctic. However, the Afrotropical (2002) and Papuan regions (2008) are both still showing a slow increase in the number of published surveys, with little sign of acceleration. The difference in relative representation of the six biogeographic regions was also noticeable, with butterfly survey efforts biased towards the Northern Hemisphere and more temperate regions. When comparing survey effort relative to described butterfly species diversity, the Palearctic and Nearctic regions were strongly overrepresented compared to the Afrotropical and Neotropical regions (Figure 4A; Appendix S1: Figure S2A), while comparing survey effort in relation to regional land area showed a particularly strong overrepresentation for the Oriental region, with the Afrotropical region strongly underrepresented (Figure 4B; Appendix S1: Figure S2B).

**FIGURE 3:**
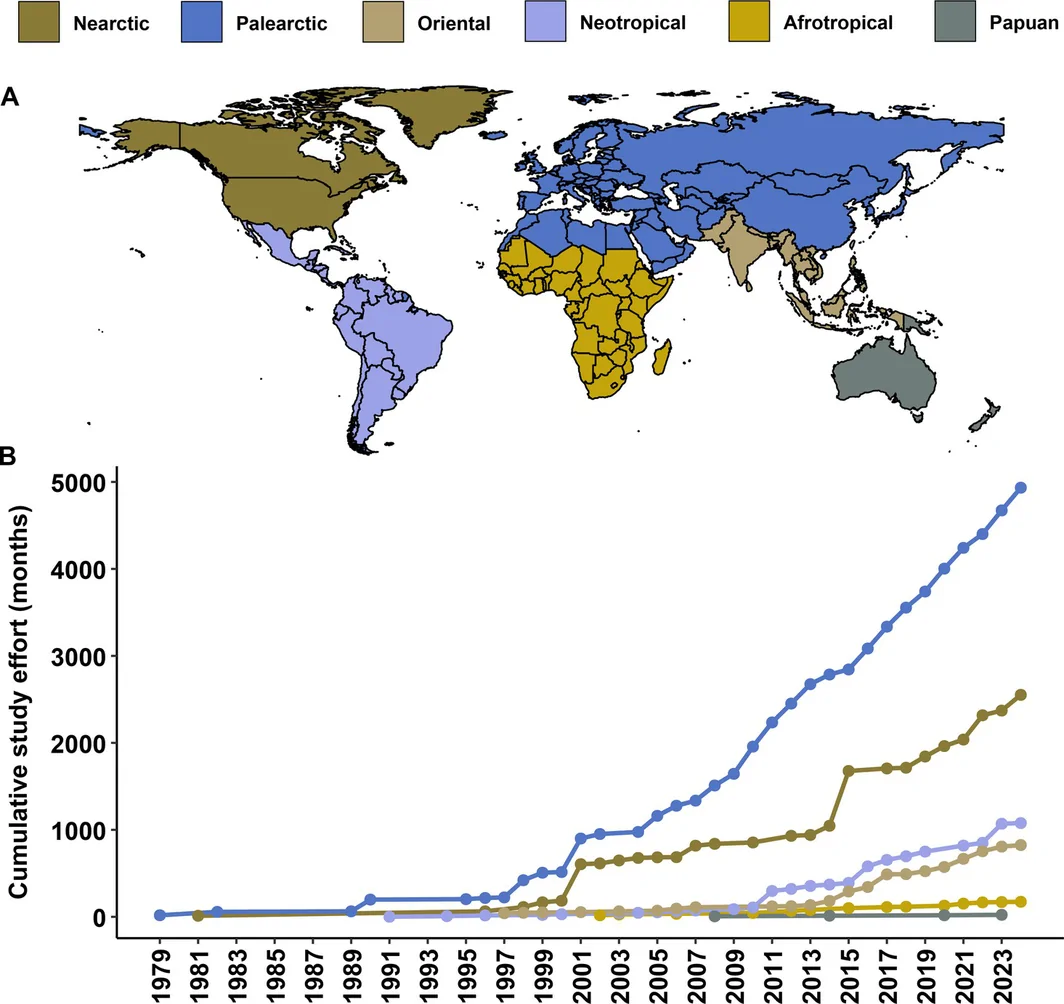
(A) Biogeographic classification of the world by countries broadly following Larsen (2005). (B) Temporal trends in the cumulative number of months of butterfly surveys across different biogeographic regions.

**FIGURE 4:**
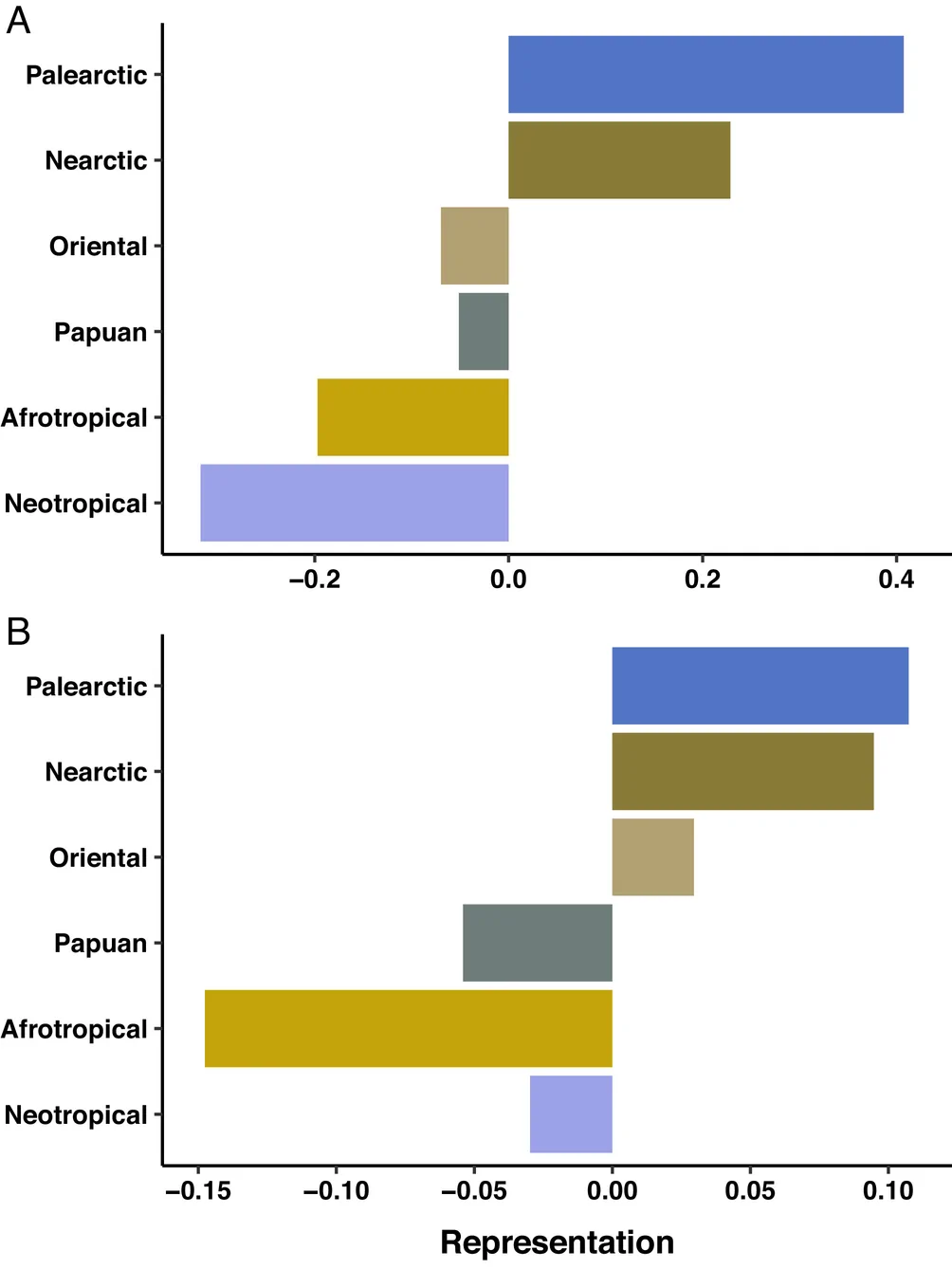
Representativeness in butterfly survey research output in different biogeographic regions relative to (A) number of described species from the region (Larsen, 2005), and (B) land area of the region. A value of zero means that the proportional number of butterfly surveys published from a region matches the relative species richness or land area of the region compared to the global output. Negative values and positive values indicate regions that are under‐ or over‐represented, respectively.

### Evolution and justification of butterfly survey protocols

The relative frequency of different butterfly survey protocol types has changed over time (Figure 5). Line transects, being the first protocol type to be used (appearing in the late 1970s) (Pollard, 1977), were still the most dominant survey protocol used, while hand netting, which emerged soon after as an alternative protocol (and has a long amateur history), initially gained considerable usage but then declined. From the mid‐1990s onwards, five new protocol types appeared over less than a decade, and the pattern of relative usage across all protocols remained relatively stable over the past two decades, with about 40% of surveys to date employing protocols other than line transects. Regardless of whether older (and well‐established) or newer protocols were employed, only a relatively small proportion of surveys justified their choice of survey methodology (14%) (Figure 6). However, two of the newer protocols (bait trapping and opportunistic recording) have a higher proportion of justification for protocol choice (Figure 6).

**FIGURE 5:**
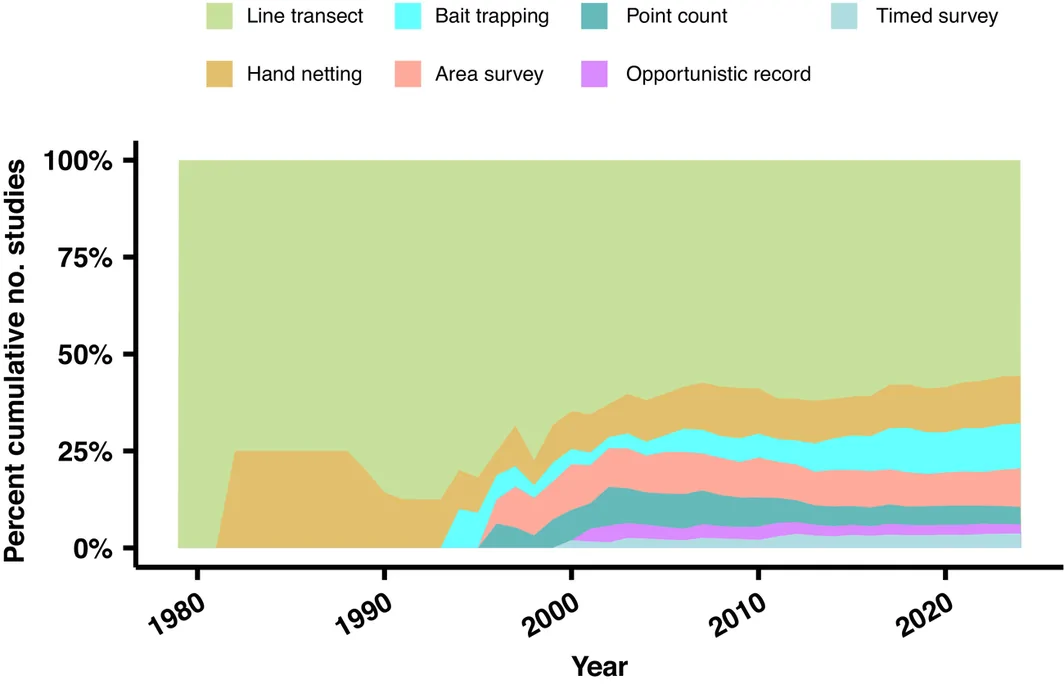
Cumulative relative use of butterfly survey protocol types overtime.

**FIGURE 6:**
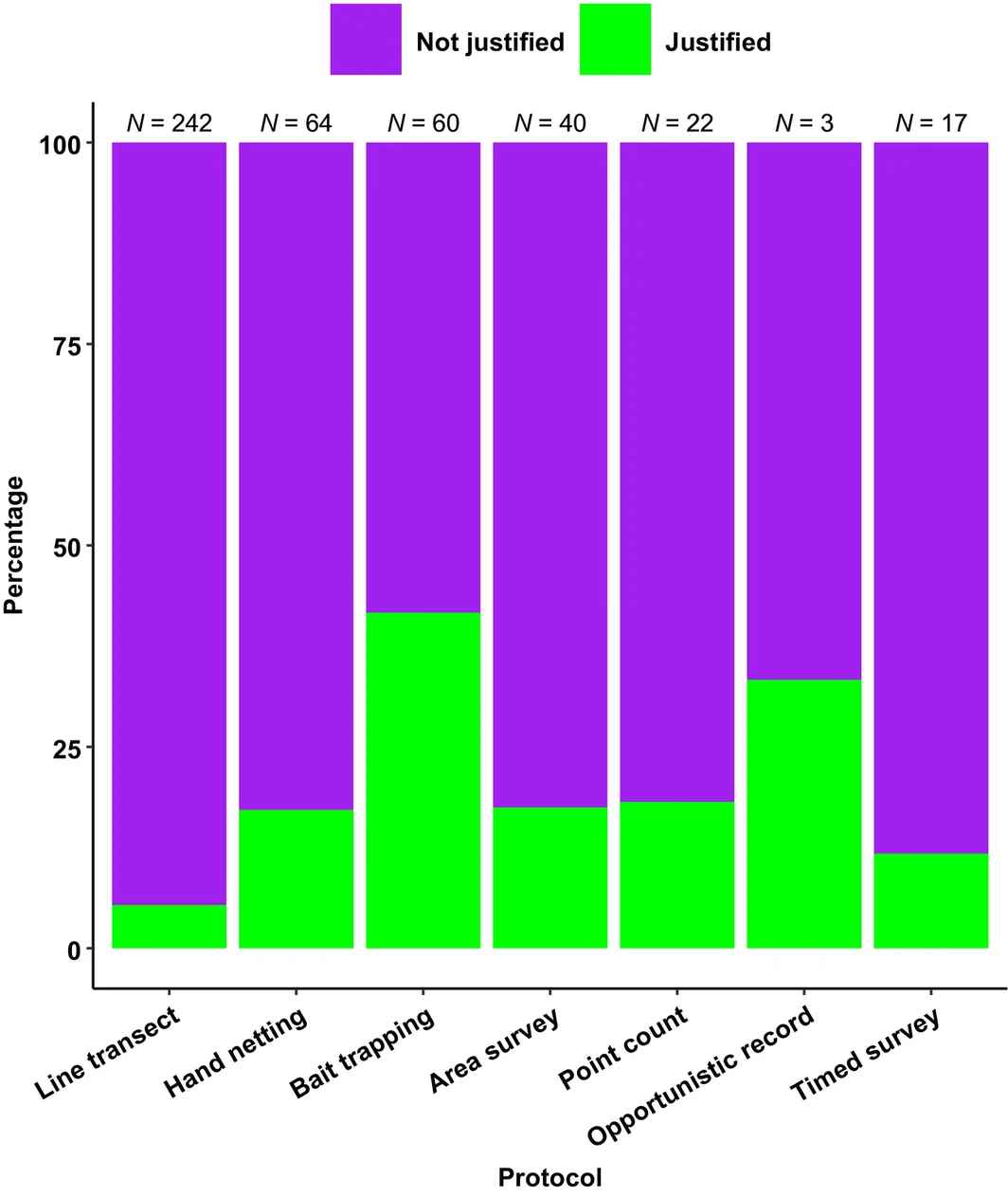
Comparison of protocol justification levels between different protocols in order of their first use.

### Determinants of butterfly survey protocol selection

Biogeographic region and study focus group (i.e., whether a study surveys butterfly biodiversity broadly, targets a group of species, or focuses on a single species) were the only variables that significantly impacted the selection of butterfly survey protocol (χ² = 171.01, df = 30, *p* < 0.001 and χ² = 63.44, df = 12, *p* < 0.001, respectively). In all biogeographic regions, the line transect protocol was the most frequently applied for butterfly surveys, except in the Neotropics where bait trapping was the most used (Figure 7A). Bait trapping was also frequently used in the Afrotropical region (Figure 7A). Hand netting had relatively high usage in the Neotropical and Oriental region (Figure 7A). Most other protocols had only limited usage in relative terms. With regard to protocol selection in relation to the study focal groups, we found a higher probability of using bait trapping when studying groups of species (e.g., fruit feeding butterflies in the family Nymphalidae), while line transects were most frequently used for studies of overall butterfly biodiversity or single species studies (Figure 7B).

**FIGURE 7:**
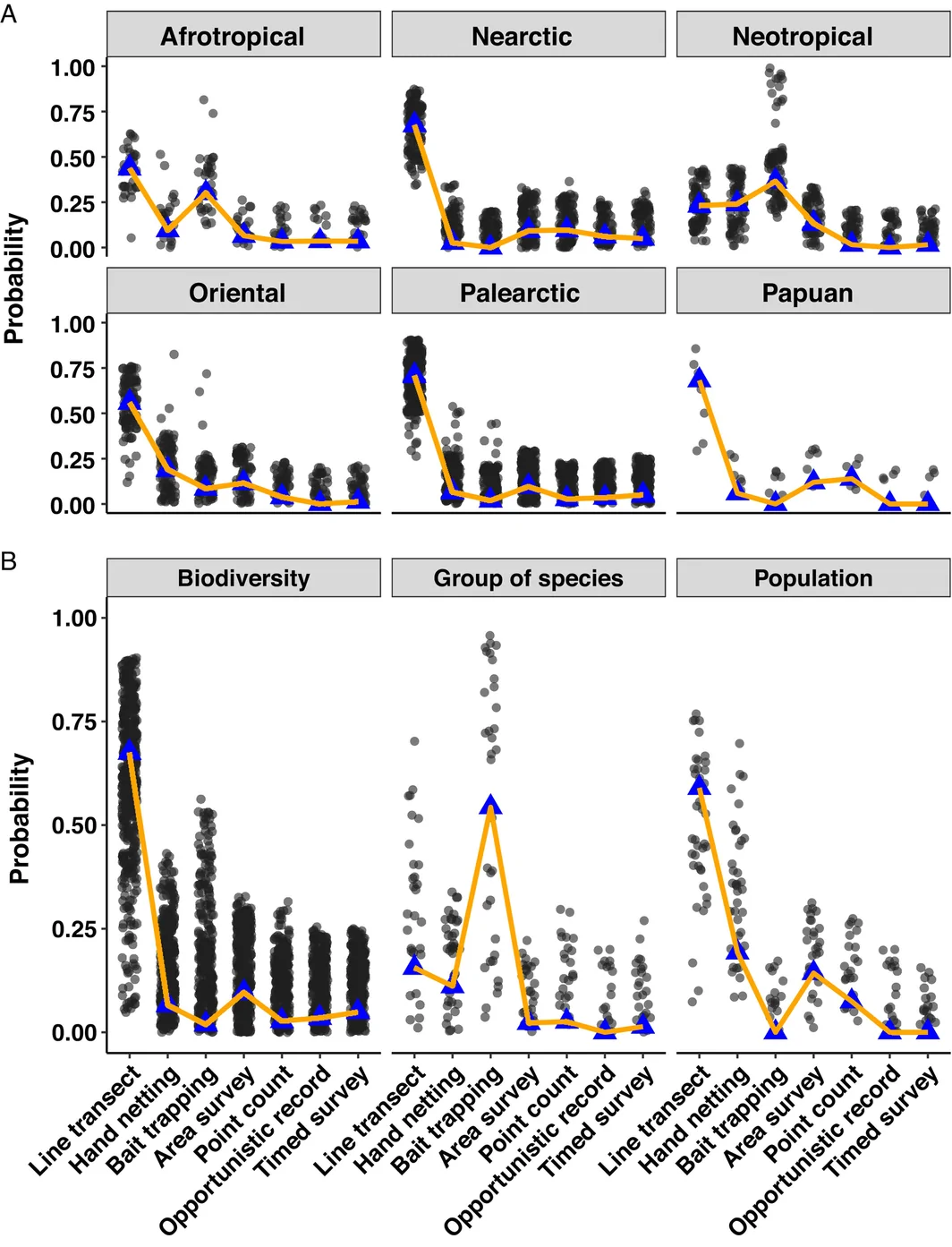
Probability of different butterfly survey protocols being selected for butterfly survey in (A) different biogeographic regions, or for different (B) study focal groups.

## DISCUSSION

### Overview of key findings

Butterfly monitoring has seen significant growth since the first systematic butterfly monitoring scheme was set up almost 50 years ago, with a corresponding increase in the diversity of protocols now being employed for butterfly surveys worldwide. However, despite this increase, efforts are still disproportionately concentrated in the Palearctic and Nearctic regions. By contrast, the Afrotropical and Neotropical regions, despite their high butterfly species diversity and the increasing environmental threats to the unique habitats in the regions, remain worryingly underrepresented. Over time, line transects (which were the first survey protocol developed and documented for systematic monitoring) have remained the dominant survey protocol, although alternative survey protocols have gained significant use from the late 1990s onwards and have stabilized in the past two decades, with nearly 40% of surveys now employing non‐line transect protocols. However, we identified a critical gap in the justification of survey protocol use, with only a small proportion (14%) of studies stating explicit reasoning for their survey protocol selection. Furthermore, studies focused specifically on method development are notably low. Addressing these gaps is essential to improving the robustness and applicability of butterfly monitoring efforts, especially in biodiversity‐rich regions where data for effective conservation planning are urgently needed, as well as underpinning the comparison of trends between different monitoring schemes (Gonzalez et al., 2023).

### Global pattern and trends in butterfly survey effort

Growth in the number of butterfly survey studies has changed considerably among regions over time. Most regions reached maximum growth from 2013 onwards. The higher growth rate in this period could be due to an increased drive to understand the global distribution of biodiversity (Meyer et al., 2015), the response of biodiversity to climate and land uses change including for butterflies (Kannan & James, 2009; Muñoz‐Galicia et al., 2023), as well as to the increasing mobilization of citizen (or community) monitoring initiatives (Prudic et al., 2017; Tosa et al., 2021). Although regions in the Global South (Neotropics, Afrotropics, Oriental and Papuan regions) showed similar growth as the regions in the Global North (Palearctic and Nearctic) with regard to the number of butterfly studies in recent years, the absolute number of studies is still much lower, and more effort is needed to match the effort already invested in the Global North. Several factors likely contribute to this geographical imbalance. As with other biodiversity monitoring schemes, butterfly research is influenced by differences in scientific infrastructure, long‐term funding availability, access to research networks, and capacity to sustain monitoring and coverage. Many countries in the Global South face difficulty maintaining such programs due to limited research resources, a shortage of specialist taxonomists, and competing societal priorities. These constraints can result in fewer long‐term datasets, despite these regions containing the highest levels of butterfly diversity. Priority regions include the Afrotropical and Papuan regions. Butterfly studies have started more recently in these regions; yet, research has not really taken off as other regions that started around the same time (Oriental and Neotropical regions), and the number of studies from these regions remains low. The Afrotropical region is particularly notable because it combines exceptionally high butterfly diversity with comparatively limited research coverage (Larsen, 2005). Along with the Neotropics, the two regions hold about two‐thirds of described butterfly diversity (12,000 species). This further shows the importance of these regions in the conservation and understanding of butterfly distribution at the global level. At the same time, this knowledge gap occurs in regions experiencing rapid environmental change (Muñoz‐Galicia et al., 2023). In the Afrotropical region, agriculture expansion, deforestation, and urbanization are major drivers of habitat loss, with pressures on natural habitat increasing substantially in recent years (Winkler et al., 2021). Without sufficient biodiversity monitoring, the impact of these changes on butterfly populations remains difficult to quantify, limiting our ability to develop effective conservation interventions. This calls for the need for environmental monitoring to assess and mitigate the impact of these changes to guide conservation efforts and policy. Despite recent promising initiatives in the Afrotropics (e.g., extension of the eBMS to Botswana, Kenya, Morocco, Nigeria, Saint Helena, South Africa, and Zambia; Aduse‐Poku et al., 2012; Edge & Mecenero, 2015; Mecenero et al., 2022; Ombugadu et al., 2023; Talavera et al., 2023), clearly more research and funding are needed (Dongmo et al., 2023).

### Evolution and justification of butterfly survey protocols

The growing need for more reliable biodiversity indices, tailored to different ecological contexts, and the increasing emphasis on adaptive management in response to environmental changes (Williams, 2011), has led to the emergence of new survey techniques. Moreover, the continued development of ecological research methods (McCallen et al., 2019) has resulted in shifts in biodiversity monitoring approaches, for example the growing use of citizen science platforms, digital imaging, environmental DNA (eDNA), automated insect monitoring systems, remote sensing, and statistical modeling approaches (Tosa et al., 2021; van Klink et al., 2022). We found similar trends in our review where the use of butterfly survey protocols has changed considerably over time. While the use of newer protocols is increasing, the line transect seems to still underpin many long‐term datasets. The widespread use of line transects could be due to their practicality, repeatability, and ability to provide standardized data across broad spatial and temporal scales. The introduction of newer protocols like point counts, area surveys, and bait trapping in the late 1990s and early 2000s shows a diversification of protocols to address the varying needs of different ecological studies and environments (e.g., Habel et al., 2022; Kral‐O'Brien et al., 2021). Indeed, some widely used biological sampling techniques can be more challenging in tropical or closed ecosystems (e.g., distance sampling; Gale et al., 2009; Pollard walks; Walpole & Sheldon, 1999), with most techniques developed in the Global North and typically for relatively open habitats. These newer protocols have shown stable usage patterns, suggesting their growing acceptance and integration into standard butterfly survey and monitoring practices.

However, we also found a widespread lack of justification for protocol choice. This raises concerns about whether survey protocols are sufficiently evaluated for their suitability, effectiveness, and efficiency before use (Whitworth et al., 2017). Furthermore, noticeably very few studies focused specifically on method development (making up about only 11% of all studies), with none from the Afrotropical or Papuan regions. Thus, ecology is lagging behind medical research approaches, where the effectiveness and value of different procedures are routinely assessed (sensu Pullin & Knight, 2001). It is particularly problematic given that different protocols could potentially yield different biodiversity estimates (Bosch et al., 2017; Whitworth et al., 2017), thereby affecting the accuracy of the data collected, and the subsequent analyses and interpretations of butterfly population trends and conservation priorities. Furthermore, without clear justifications, it becomes difficult to assess whether monitoring efforts are optimized for their intended purpose or whether they are being adopted out of convention. There is, therefore, a clear need for further comparisons of different survey protocols in different environments to assess their suitability (Pellet et al., 2012), as well as comparisons of the estimates produced across survey protocols (Barkmann et al., 2023) and monitoring schemes (Roy et al., 2015). In addition, researchers should be critical in selecting survey protocols, considering suitability, accuracy, and effectiveness, while clearly justifying and communicating their decisions.

We note that opportunistic observations might have been underrepresented in our review. Opportunistic observations are one of the earliest protocols used for recording biodiversity (e.g., field observations), with a long history (Pocock et al., 2015). The few studies that used this protocol identified in our review could be because the term “opportunistic observations” is more recent terminology, associated with the establishment of wider biological recording (Pocock et al., 2015), and often associated with biological atlases describing the distribution of biodiversity in a region. Protocols that are easy to implement, like reporting opportunistic observations, are useful entry points for a broader participation of amateurs, citizens, and communities in biodiversity monitoring (Brown & Williams, 2018), which could be particularly valuable in regions with limited structured monitoring (Tulloch et al., 2013). Indeed, huge volumes of opportunistic observations are now being generated by the increasing popularity of global citizen science platforms like iNaturalist.org (Tosa et al., 2021). In addition, datasets collected through opportunistic observations are increasingly being used to assess biodiversity trends (Boyd et al., 2023). Although studies using opportunistic data often have a broad taxonomic focus and rarely assess abundance (Pagel et al., 2014), opportunistic observations are less relevant in systematic monitoring but could be increasingly important for integrated future butterfly survey efforts (Kühl et al., 2020).

### Choosing butterfly survey protocols

The lack of consensus on a universally adopted survey protocol could be because protocol choice is influenced by many factors such as ecological context and regional differences in species composition and behavior (Habel et al., 2022; Kral‐O'Brien et al., 2021). Similarly, the choice of monitoring protocol could also be influenced by the objective of the survey (Jetz et al., 2019). Butterfly monitoring may aim to quantify species richness and community composition, abundance and temporal trends, document species distributions and occupancy patterns, detect rare or specialist taxa, or assess responses to environmental change and conservation interventions. These differing objectives might not necessarily require equivalent survey approaches (van Swaay et al., 2008). For example, protocols designed for long‐term population trend estimation will require high repeatability, standardization, and consistent sampling effort through time (Sparrow et al., 1994), making fixed route transects, timed surveys, area survey, or point counts particularly valuable. In contrast, inventories focused on maximizing species detection and documenting community richness may benefit from more flexible or complementary protocols such as area surveys, bait trapping, opportunistic observations, or hand netting (Checa et al., 2019), which can improve detectability of cryptic, canopy‐dwelling, or behaviorally specialized species. Similarly, large‐scale distribution or occupancy assessments may rely more heavily on opportunistic observations because of their broad spatial coverage, despite lower control over sampling effort (van Strien et al., 2013).

Amid this diversity of methodological choices, line transects are the most widely used protocol over time, a pattern that could possibly be due to their ability to reconcile two competing demands in biodiversity monitoring: achieving high spatial coverage while maintaining repeatability and standardization. By following fixed routes, transects allow observers to systematically sample across heterogeneous habitats, increasing detection probability and producing data that are both spatially representative and comparable over time, making them particularly valuable for long‐term monitoring programs (Isaac et al., 2011; Pellet et al., 2012; Pollard & Yates, 1993; Videvall et al., 2016). However, the reliance of line transects on real‐time detection and identification places a substantial cognitive burden on observers, especially in species‐rich assemblages where rapid identification among similar morphotaxa is required, potentially leading to increased identification error and decreased detection efficiency (Attiwilli et al., 2024). Furthermore, their application is constrained by landscape accessibility, thereby creating potential biases in habitat representation, particularly in dense forests or rugged terrain where some of the most diverse butterfly communities occur. In such contexts, point counts (e.g., Henry et al., 2015) offer a potentially more practical alternative: By retaining the principles of standardization and repeatability while removing the need for movement, they provide a structured approach that is more feasible in inaccessible or structurally complex habitats. Although point counts capture a more spatially restricted snapshot and still depend heavily on observer expertise, their stationary nature may reduce cognitive load and allow more careful identification, partially offsetting some limitations of transects. Beyond these two, other protocols each address specific weaknesses but introduce distinct trade‐offs that further emphasize the absence of a universally optimal approach. Area and timed surveys increase flexibility by allowing observers to concentrate effort in resource‐rich or biodiversity hotspots, which can improve detection and identification in complex systems (Attiwilli et al., 2024), yet this flexibility often comes at the cost of reduced standardization and comparability. Hand netting enhances taxonomic accuracy by enabling close inspection, though it is labor‐intensive and limited in spatial extent (Checa et al., 2019), making it less suitable for large‐scale monitoring. Opportunistic observations, particularly those generated through citizen science, can achieve exceptionally broad spatial coverage, and contribute valuable occurrence data, but their lack of controlled effort and observer consistency constrains their utility for rigorous quantitative assessments (van Strien et al., 2013). Bait trapping extends sampling into otherwise inaccessible areas and across vertical forest strata, thereby complementing visually based surveys and expanding ecological coverage, but its reliance on baits inherently biases sampling towards feeding guilds (Checa et al., 2019; Freitas et al., 2021), and can only be implemented where there is high diversity of fruit‐feeding butterflies (e.g., Freitas et al., 2021; Pérez et al., 2017). Taken together, these trade‐offs could imply that the widespread adoption of line transects is not because they are universally superior, but because they offer a pragmatic and effective compromise between coverage, consistency, and standardization in many monitoring contexts. However, because movement can be constrained in highly diverse or logistically challenging environments, integrating complementary approaches such as point counts may be more appropriate in such contexts (Henry et al., 2015). Ultimately, these findings reinforce that survey design should involve careful alignment between protocol choice, ecological conditions, and study objectives to balance spatial representativeness, identification reliability, and logistical feasibility, thereby ensuring robust and comparable assessments of butterfly diversity and population change across regions and scales.

Regardless of which protocol is adopted, three conditions are critical for its effective application and for enabling meaningful comparisons among monitoring schemes. First, the protocol should reliably capture butterfly richness and abundance within the target habitat. Second, survey effort should be standardized or explicitly accounted for (e.g., through time, distance, sampling area, or detection correction) to reduce sampling bias. Third, protocols should employ consistent sampling procedures, metadata recording, and reporting standards that facilitate data harmonization and integration across regions and long‐term monitoring programs (e.g., Darwin core standards; Wieczorek et al., 2012). These considerations are essential for generating robust datasets that can support regional assessments while also contributing to broader global conservation evaluations (e.g., the Global Butterfly Index; Riva et al., 2026).

Another important consideration is how data generated from different butterfly monitoring protocols can be integrated for broader assessments of population and community trends. Differences among protocols can affect estimates of abundance through variation in survey effort, detectability, sampling frequency, observer effects, and spatial coverage (Pellet et al., 2012). As a result, direct comparisons of raw counts obtained using different methods may be challenging without accounting for these sources of variation. However, protocol differences do not necessarily prevent the integration of monitoring data because they might complicate comparisons, but such differences are less problematic if they produce unbiased and reliable abundance estimates (Jetz et al., 2019). In this context, the key consideration is whether different methods reliably capture variation in abundance rather than whether they employ identical sampling procedures. If different protocols provide reliable and consistent estimates of relative abundance, the resulting population metrics are expected to be strongly correlated, allowing meaningful comparisons and integration across monitoring schemes (Isaac et al., 2020). Previous studies have shown that abundance estimates derived from different survey approaches, such as Pollard transects and area‐time counts, can have strong relationships and may therefore be suitable for generating comparable trend indicators when methodological differences are appropriately accounted for (Barkmann et al., 2023). Furthermore, recent advances in statistical modeling facilitate the integration of datasets collected under different protocols. Hierarchical models, occupancy frameworks, and approaches that explicitly account for detectability and sampling effort can help harmonize data from multiple sources, allowing estimation of population and community trends across larger spatial and temporal scales (Henry et al., 2025; Schmucki et al., 2015). Although protocol‐specific biases should be carefully evaluated, the use of different monitoring approaches does not necessarily preclude their combined use in broader analyses. Instead, employing multiple complementary protocols may enhance monitoring coverage and increase the spatial representativeness of biodiversity assessments, provided that methodological differences are acknowledged and incorporated into the analytical framework.

## CONCLUSIONS

Our review shows that butterfly survey research remains heavily concentrated in the temperate Global North, with the Afrotropical, Neotropical, and Papuan regions particularly underrepresented despite their exceptionally high butterfly diversity. This mismatch is troubling, especially considering escalating land use pressures, rapid agricultural expansion, and ongoing deforestation across these regions. Without systematic monitoring, the true impacts of these threats remain poorly quantified, risking substantial underestimation of biodiversity loss. Addressing these deficits requires urgent investment in long‐term monitoring programs, targeted ecological research, and sustained capacity building in tropical regions. Filling these spatial gaps is essential not only for local conservation planning but also for achieving a genuinely global understanding of butterfly population trends and responses to environmental change.

Although we documented a broad and increasing diversity of methods, and the dominance of line transects over several decades, very few studies evaluated their chosen protocols or provided explicit rationale for their selection. This methodological inconsistency poses a major challenge: Survey design strongly influences detectability, abundance estimates, and ultimately the conservation conclusions drawn from data (Schmidt et al., 2023). In regions already lacking baseline information, especially in the tropics, reliance on untested or convention‐based protocols risks misrepresenting population dynamics, overlooking vulnerable species, and misdirecting conservation action. To strengthen the reliability and interpretability of butterfly data globally, researchers must prioritize empirical assessment of method performance across ecological contexts, including comparisons of precision, efficiency, and representativeness. These are crucial steps towards developing evidence‐based, standardized protocols tailored to habitat types and monitoring goals and allow producing high‐quality, comparable datasets capable of informing robust regional and global conservation interventions.

## AUTHOR CONTRIBUTIONS

**Joseph Izang Ibrahim:** Writing—initial draft and editing; visualization; validation; software; methodology; investigation; formal analysis; data curation; conceptualization. **Sofie Spatharis:** Writing—review and editing; visualization; validation; software; methodology; investigation; formal analysis; conceptualization. **Oskar Brattström:** Writing—review and editing; visualization; validation; software; methodology; investigation; formal analysis; conceptualization. **Rob Cooke:** Writing—review and editing; visualization; validation; software; investigation; formal analysis. **David B. Roy:** Writing—review and editing; visualization; validation; software; investigation; formal analysis.

## CONFLICT OF INTEREST STATEMENT

The authors declare no conflicts of interest.

## Supporting information


Appendix S1.

## Data Availability

The list of publications that were analyzed and all relevant information that was extracted from each publication is available in Ibrahim et al. (2026) in Dryad at https://doi.org/10.5061/dryad.8gtht773g.
