# Supplementary material for: Biases in butterfly survey effort and patterns of protocol used across biogeographic regions
Source: Ecol Appl. 2026 Oct 4;36(7):e70326. doi: 10.1002/eap.70326 (PMC13635469; doi:10.1002/eap.70326)
Supplement: Supplementary file 1 [file EAP-36-e70326-s001.pdf]

Appendix S1

Biases in butterfly survey effort and patterns of protocol used across biogeographic regions

Joseph Izang Ibrahim, Sofie Spatharis, Rob Cooke, David B. Roy, and Oskar Brattström

Ecological Applications

Box S1. Search string.

TS=((“survey protocol*” OR “sampling protocol*”OR “monitoring protocol*” OR “assessment protocol*” OR “biodiversity monitoring” OR “population survey*” OR “biodiversity survey*” OR “biodiversity assessment” OR “biodiversity monitoring” OR “population monitoring” OR “population assessment” OR “butterfly richness” OR “butterfly abundance” OR “butterfly biodiversity”) AND (butterfl* OR rhopalocera OR hesperidine OR papilionodae OR zygaenidae OR ((day OR diurnal) AND moth))

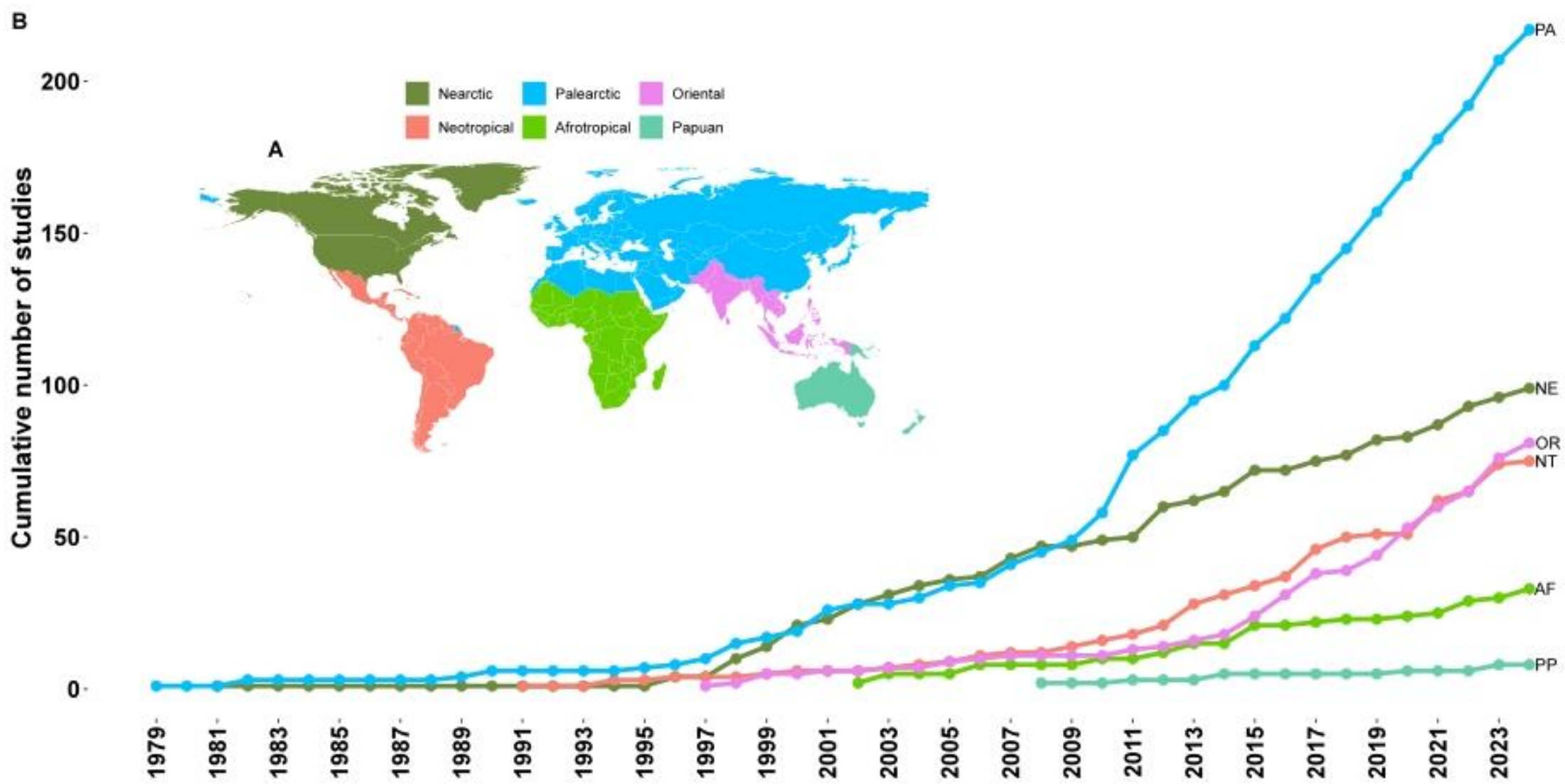

Figure S1. (A) Biogeographic classification of the world by countries following Larsen (2005). (B) Temporal trends in the cumulative number of butterfly survey studies across different biogeographic regions (PA = Palearctic, NE = Nearctic, OR = Oriental, NT = Neotropical, AF = Afrotropical and PP = Papuan regions).

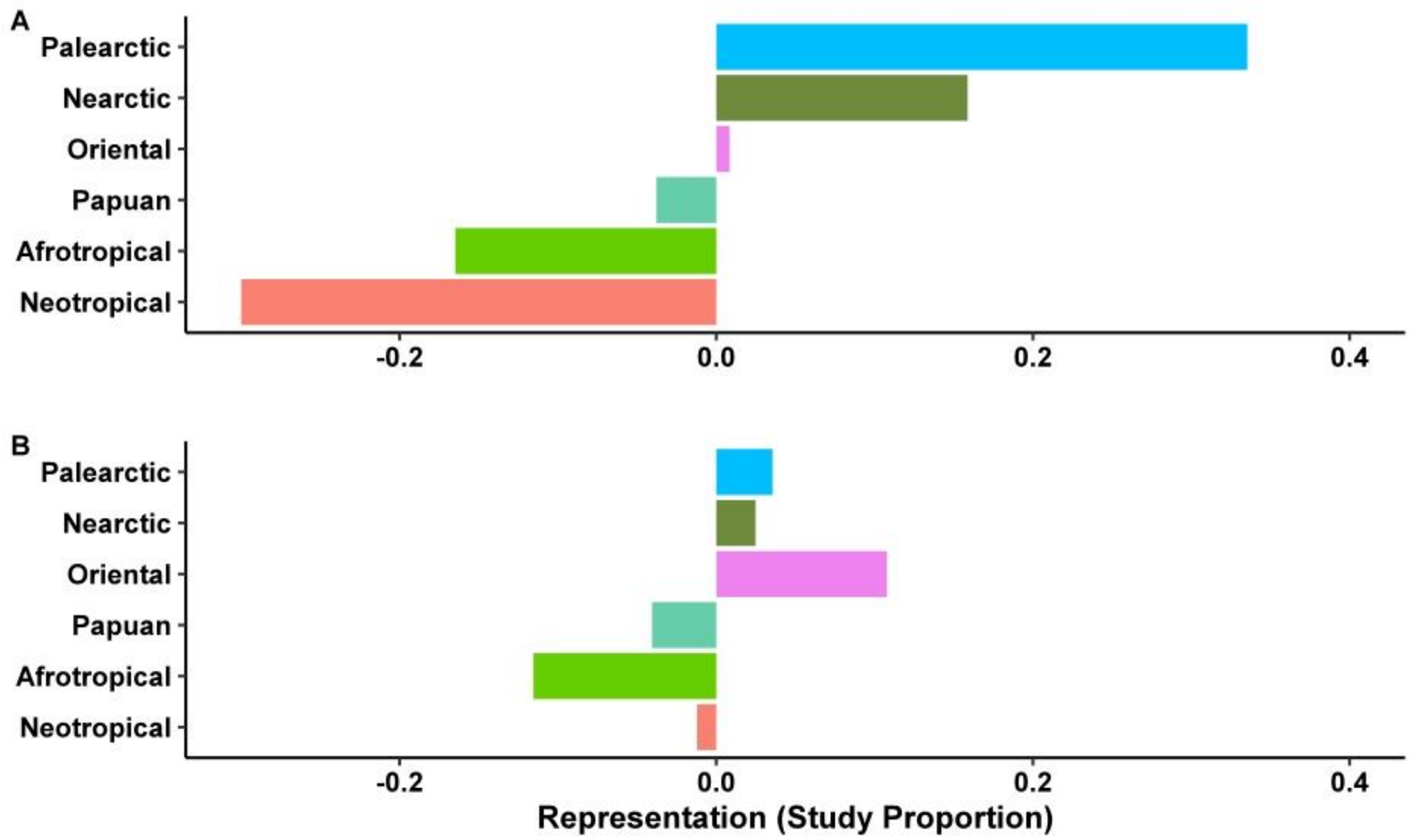

Figure S2. Representativeness in butterfly survey research output (number of studies) in different biogeographic regions relative to (A) number of described species from the region (Larsen 2005), and (B) land area of the region. A value of zero means that the proportional number of butterfly surveys published from a region matches the relative species richness or land area of the region compared to the global output. Negative values and positive values indicate regions that are under- or over-represented respectively.

Reference

Larsen, T. B. (2005). Butterflies of West Africa: text volume (pp. 270). Apollo Books.
